# COVID-19 Vaccine Tweets After Vaccine Rollout: Sentiment–Based Topic Modeling

**DOI:** 10.2196/31726

**Published:** 2022-02-08

**Authors:** Luwen Huangfu, Yiwen Mo, Peijie Zhang, Daniel Dajun Zeng, Saike He

**Affiliations:** 1 Fowler College of Business San Diego State University San Diego, CA United States; 2 Center for Human Dynamics in the Mobile Age San Diego State University San Diego, CA United States; 3 The State Key Laboratory of Management and Control for Complex Systems Institute of Automation Chinese Academy of Sciences Beijing China; 4 University of Chinese Academy of Sciences Beijing China

**Keywords:** COVID-19, COVID-19 vaccine, sentiment evolution, topic modeling, social media, text mining

## Abstract

**Background:**

COVID-19 vaccines are one of the most effective preventive strategies for containing the pandemic. Having a better understanding of the public’s conceptions of COVID-19 vaccines may aid in the effort to promptly and thoroughly vaccinate the community. However, because no empirical research has yet fully explored the public’s vaccine awareness through sentiment–based topic modeling, little is known about the evolution of public attitude since the rollout of COVID-19 vaccines.

**Objective:**

In this study, we specifically focused on tweets about COVID-19 vaccines (Pfizer, Moderna, AstraZeneca, and Johnson & Johnson) after vaccines became publicly available. We aimed to explore the overall sentiments and topics of tweets about COVID-19 vaccines, as well as how such sentiments and main concerns evolved.

**Methods:**

We collected 1,122,139 tweets related to COVID-19 vaccines from December 14, 2020, to April 30, 2021, using Twitter’s application programming interface. We removed retweets and duplicate tweets to avoid data redundancy, which resulted in 857,128 tweets. We then applied sentiment–based topic modeling by using the compound score to determine sentiment polarity and the coherence score to determine the optimal topic number for different sentiment polarity categories. Finally, we calculated the topic distribution to illustrate the topic evolution of main concerns.

**Results:**

Overall, 398,661 (46.51%) were positive, 204,084 (23.81%) were negative, 245,976 (28.70%) were neutral, 6899 (0.80%) were highly positive, and 1508 (0.18%) were highly negative sentiments. The main topics of positive and highly positive tweets were planning for getting vaccination (251,979/405,560, 62.13%), getting vaccination (76,029/405,560, 18.75%), and vaccine information and knowledge (21,127/405,560, 5.21%). The main concerns in negative and highly negative tweets were vaccine hesitancy (115,206/205,592, 56.04%), extreme side effects of the vaccines (19,690/205,592, 9.58%), and vaccine supply and rollout (17,154/205,592, 8.34%). During the study period, negative sentiment trends were stable, while positive sentiments could be easily influenced. Topic heatmap visualization demonstrated how main concerns changed during the current widespread vaccination campaign.

**Conclusions:**

To the best of our knowledge, this is the first study to evaluate public COVID-19 vaccine awareness and awareness trends on social media with automated sentiment–based topic modeling after vaccine rollout. Our results can help policymakers and research communities track public attitudes toward COVID-19 vaccines and help them make decisions to promote the vaccination campaign.

## Introduction

### Background

COVID-19 vaccines are one of the most effective preventive strategies for containing the pandemic and restoring normal life [1]. The outcomes of this strategy highly depend on vaccination coverage, wherein herd immunity requires at least 70% of the population to be immune, depending on how contagious the COVID-19 variant in question is and how effective the vaccine is [2]. However, such a high rate of vaccination cannot be reached without the cooperation of the general public [3-5]. In general, there are a variety of factors that may negatively impact how the public perceives and reacts to these vaccines. Such barriers may stem from conspiracy theories [6], general hesitancy toward vaccines [4], and doubts regarding new mRNA vaccine technologies [7]. Infodemic management, that is, managing information overload, including false or misleading information [8], should be used during the COVID-19 pandemic, by listening to community concerns, preventing the spread of misleading information [9], and examining the human factors contributing to COVID-19 transmission [10]. Thus, to promote vaccine awareness and facilitate vaccine rollout, it is imperative to gain a timely understanding of the public’s attitude toward vaccination and develop tailored communication strategies to address their concerns.

Generally, characterizing public vaccine attitudes as part of public health surveillance can be achieved via social media–based text mining or other traditional methodologies, such as conducting surveys or experiments. Social media–based text mining has become increasingly popular because of its effectiveness and efficiency; the major merit of this big data analysis is that it addresses several of the limitations of traditional methodologies, such as the inability to track real-time trends [4,11]. Public health monitoring on social media has proven to be a powerful tool for analyzing public health discussions on a variety of topics, such as pandemics and vaccination [12-24]. Such work has been conducted for the COVID-19 pandemic (Multimedia Appendix 1). However, because of the rapid COVID-19 vaccine rollout, dedicated social media–based sentiment analysis studies on vaccine awareness have just started to emerge [3,22-24]. Some of these studies [3,22] relied on natural language processing techniques to conduct large-scale sentiment analysis about vaccines, while others [23,24] investigated vaccination hesitancy using manual content analysis, but overall, these studies lacked either the capability to automatically track public attitudes (in manual content analysis) or a comprehensive view of both topics and associated sentiments. Furthermore, exploring the public sentiment and concern evolution throughout the current vaccination campaign may allow policymakers to make timely and informed decisions to encourage vaccination.

### Study Objectives

We aimed to combine sentiment analysis and topic modeling in order to address the following research questions: What are the general sentiments on COVID-19 vaccines? What are the topics that shape the sentiments? How do concerns (ie, topics with negative sentiments) evolve over time?

## Methods

### Data Collection

**Figure 1:**
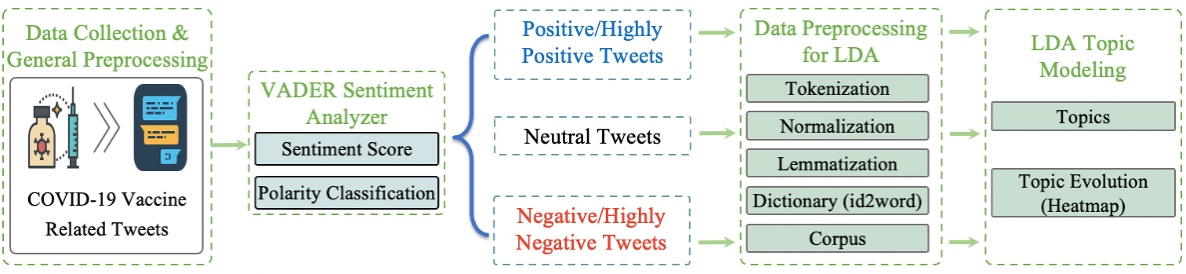
Data processing workflow. LDA: latent Dirichlet allocation; VADER: Valence Aware Dictionary for Sentiment Reasoning.

We collected COVID-19 vaccine–related tweets containing a variety of predefined hashtags, including #CovidVaccine, #GetVaccinated, #covid19vaccine, #vaccination, #AstraZeneca, #Johnson & Johnson, #Pfizer and #Moderna, from December 14, 2020 (after the first COVID-19 vaccine in the world was approved) to April 30, 2021. We collected 1,122,139 tweets (Table 1). To avoid data redundancy, we removed retweets and duplicate tweets, and we focused on tweets in English (Figure 1). After data preprocessing, the data set contained 857,128 tweets.

**Table 1:** Tweet hashtags.

Hashtag	Tweets (N=1,122,139), n
#CovidVaccine	345,537
#GetVaccinated	73,817
#covid19vaccine	130,043
#vaccination	132,327
#AstraZeneca	126,954
#Johnson & Johnson	211,731
#Pfizer	61,979
#Moderna	39,751

### Sentiment Analysis

We used the Valence Aware Dictionary for Sentiment Reasoning (VADER) lexicon for analysis. During preprocessing, we did not remove the hashtag content because it often contained meaningful information such as the brand of the vaccine. VADER is a rule–based sentiment analysis tool that has been proven to perform as well as or even better than other sentiment analysis tools on social media texts in most cases, since it is specifically attuned to sentiments expressed on social media [25]. Generally, VADER produces 4 scores: positive, neutral, negative, and compound scores. Positive, neutral, and negative scores each represent the proportion of words that fall into the given category. The compound score is calculated by summing the valence scores of each word in the lexicon, adjusting the value according to heuristic rules, and normalizing between −1 and +1 [25]. The compound score is a useful metric for measuring the sentiment of each given text in a single dimension.

We classified each tweet into 1 of 5 groups (Table 2), based on compound, positive, and negative score distributions—highly positive (compound score >0.001 and positive sentiment score >0.5), positive (compound score >0.001 and positive sentiment score <0.5), highly negative (compound score <0.001 and negative sentiment score >0.5), and negative (compound score <0.001 and negative sentiment score <0.5), and neutral (if none of the conditions was satisfied).

**Table 2:** Sentiment polarity examples.

Sentiment polarity	Example
Highly positive	“thank god vaccination vaccinessavelives vaccineswork”
Positive	“it s an exciting day with the arrival of the first coronavirusvaccine it gives me great hope for 2021 covid19vaccine”
Highly negative	“it s fake you re all stupid covidvaccine”
Negative	“how do we know that after 6 9 months there are no adverse effects of the vaccine or that it s ineffective and what s the response if in the event these emergency approvals have larger ramifications any mechanism being put together covid_19 covid19vaccine”
Neutral	“help is on the way 1st doses of covid19vaccine arrived in north carolina initial vaccine supply is limited and will go to a small number of public health and hospital workers at high risk of exposure more doses are on the way but until then practice your 3ws”

### Topic Modeling

Latent Dirichlet allocation (LDA), as a popular and well-established approach for topic analysis [26], is a three-level hierarchical Bayesian model that relies on the bag-of-words model [27]. LDA generates a probability distribution for the text corpus; it assumes that each topic can be characterized by a distribution of words. The number of topics is a key parameter of the LDA model. To prevent the misclassification of other topics into vaccine and nonvaccine topics, we removed some vaccine-related keywords, including “vaccine,” “vaccines,” “vaccination,” “covidvaccine,” and “covid.” This data preprocessing decision is also well supported by experimental results, which suggested that up to 96% of tweets were classified into one main topic with less meaningful information without removal of specific words.

**Figure 2:**
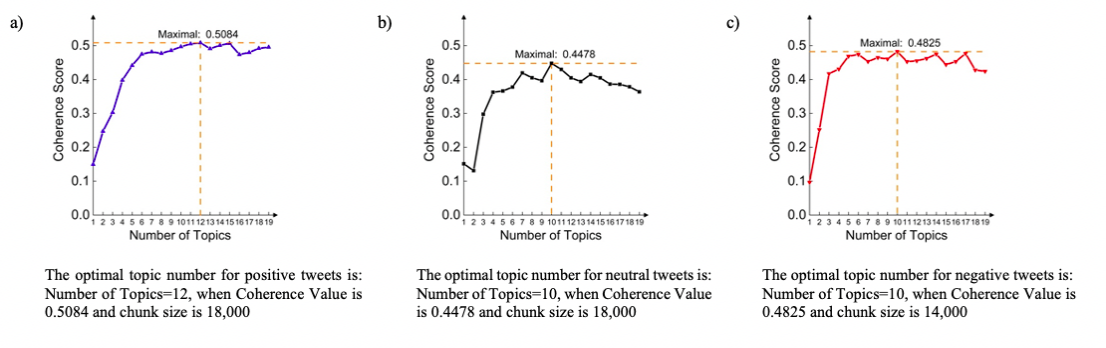
Model performance for topic numbers for (a) positive, (b) neutral, and (c) negative tweets.

To determine the optimal number of topics with favorable model performance, we used a coherence score; however, because the number of samples for highly positive and negative groups were small, we combined positive and highly positive groups (into a positive group) and negative and highly negative groups (into a negative group). Then, we applied topic modeling algorithms on 3 groups: positive, neutral, and negative. We used the topic coherence value to measure the modeling performance. Since the data set was very large, the experiments were run under the server environment with C5 computing type series IV 64-core CPU and 128 GB RAM. Then, based on the performance, we selected the optimal number of topics for each polarity group. The optimal topic numbers for positive, neutral, and negative were 12, 10, and 10, respectively (Figure 2).

## Results

### Sentiment Analysis

**Figure 3:**
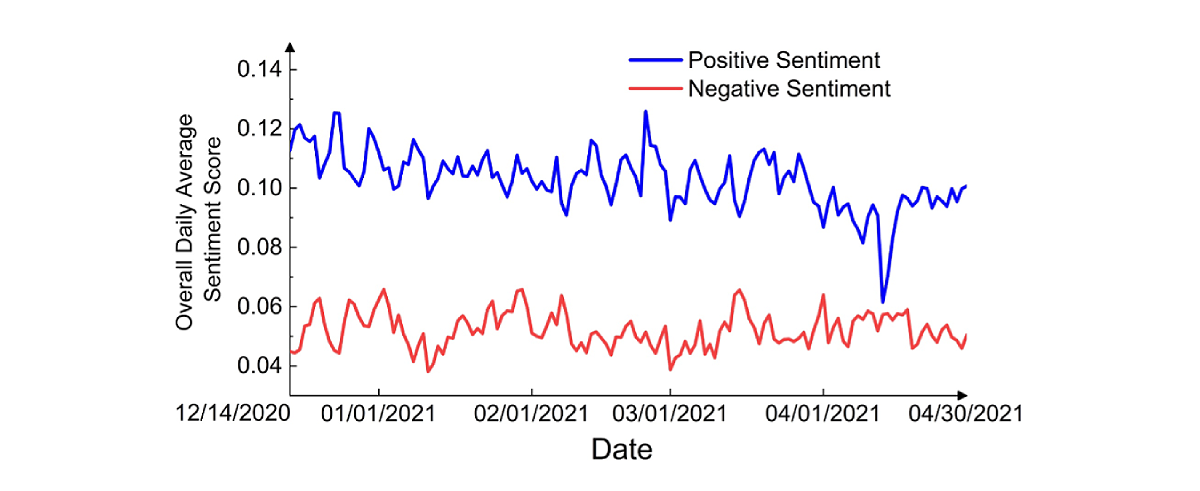
Overall daily average sentiment score.

**Figure 4:**
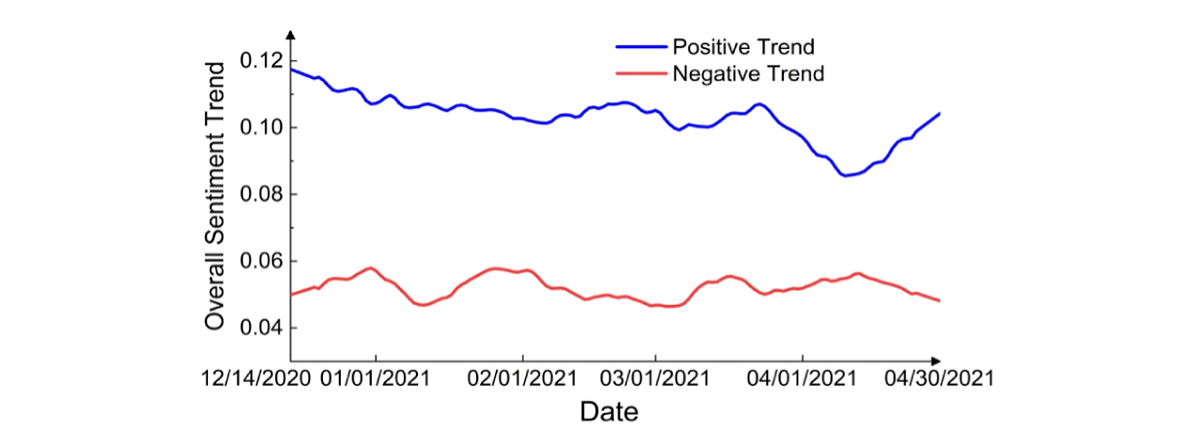
Overall sentiment trend.

Overall, positive sentiment was stronger than negative sentiment (Figure 3 and Figure 4). Notably, there was a sharp decline in the positive score around April 13, 2020 (Figure 3), which appeared to coincide with news released on that date: The US Federal Drug Administration (FDA) and Centers for Disease Control (CDC) called for a pause on the use of the Johnson & Johnson vaccine after discovering “extremely rare” cases of blood clots [28], and the number of tweets about the Johnson & Johnson vaccine peaked, reaching 23,729 tweets, which affect the average sentiment.

**Figure 5:**
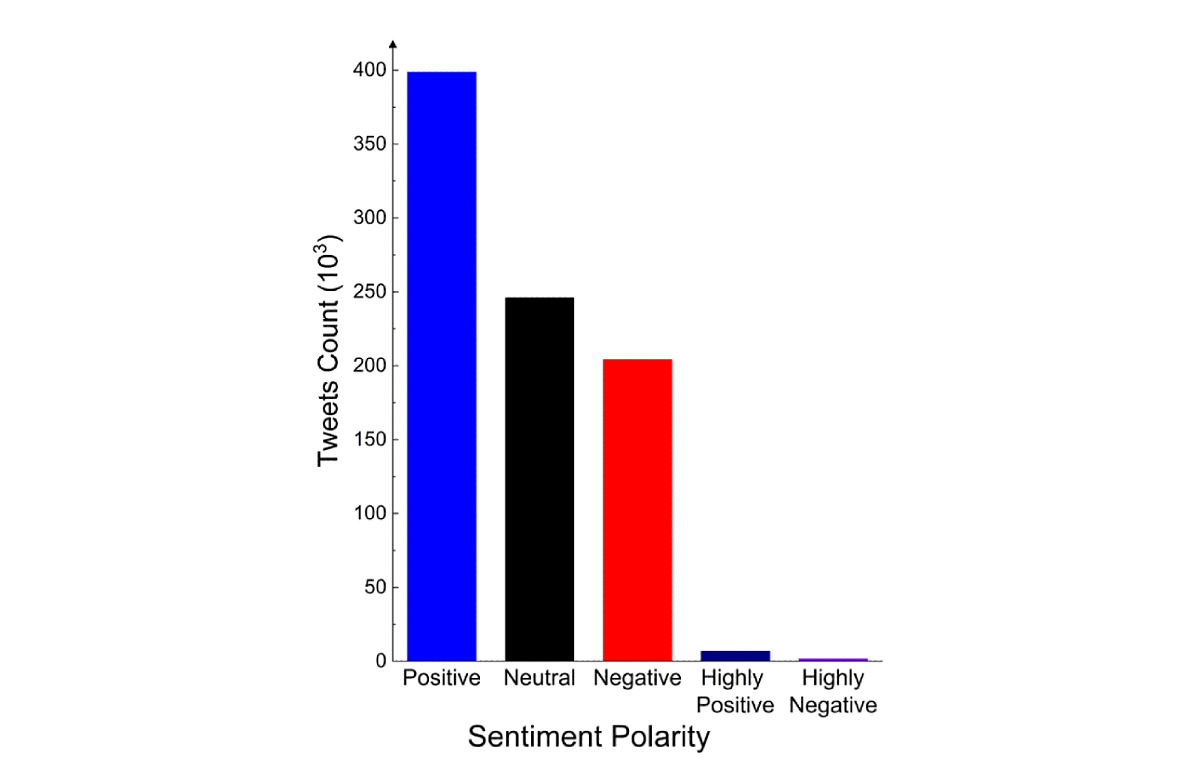
Sentiment polarity category distribution.

There were 6899 highly positive tweets, 398,661 positive tweets, 245,976 neutral tweets, 204,084 negative tweets, and 1508 highly negative tweets (Figure 5).

**Figure 6:**
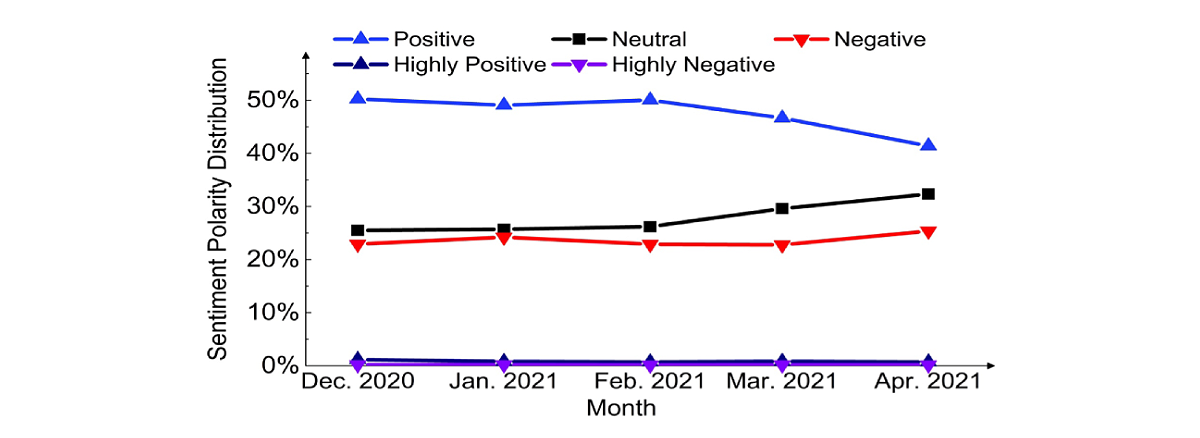
Sentiment polarity distribution by month.

The percentage of negative sentiments was stable (Figure 6), but the percentage of positive sentiments decreased by month, and the percentage of neutral sentiments increased by month. Positive sentiment likely decreased due to the pause in the use of the Johnson & Johnson and AstraZeneca vaccinations in late March and April 2021 [28]. The neutral sentiment trend moved opposite to the positive sentiment trend.

**Figure 7:**
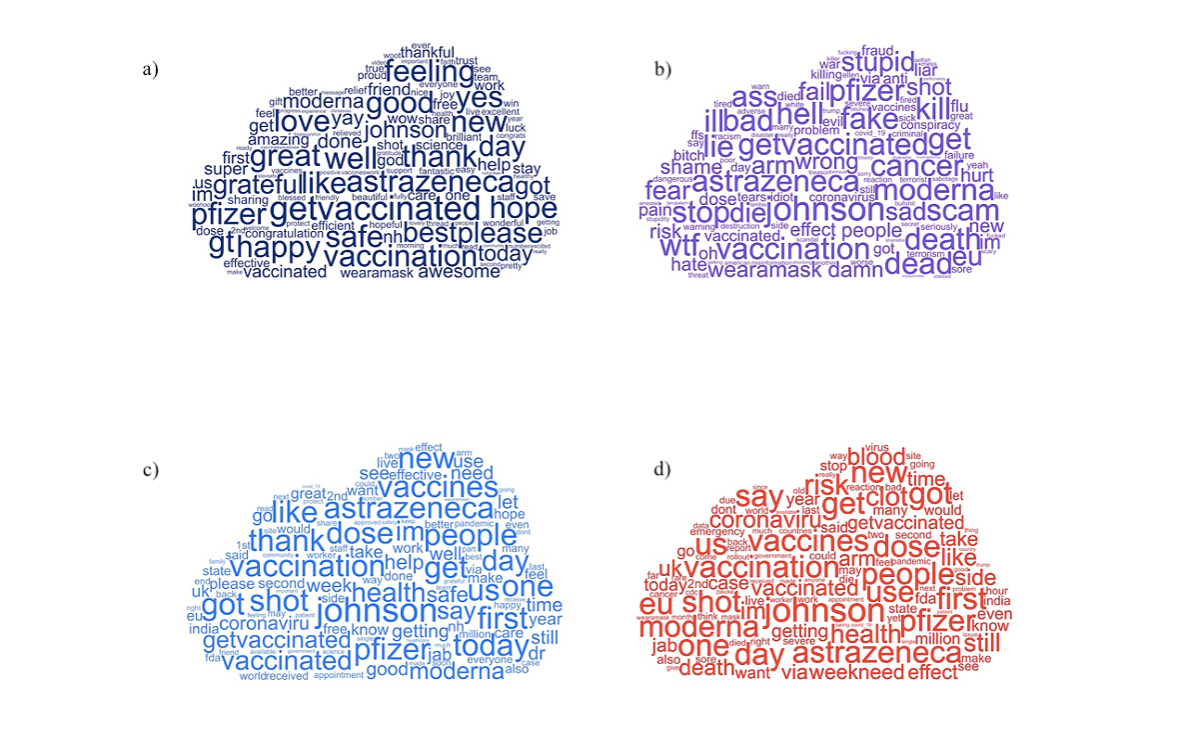
Common words for (a) highly positive, (b) highly negative, (c) positive, and (d) negative tweets.

Figure 7 shows word clouds with profanities removed for highly positive, highly negative, positive, and negative tweets. Except for “vaccine” and “COVID,” which exhibited the highest frequency, the most common positive words in the highly positive group were “great,” “happy,” and “love”; the most common negative words in the highly negative group were “kill,” “bad,” and “death”; the most common positive words in the positive group were “thank,” “like,” and “health”; and the most common negative words in the negative group were “death,” “clot,” and “risk.”

Additionally, the names of COVID-19 vaccine manufacturers *Johnson & Johnson* and *AstraZeneca* exhibited a high frequency in the negative groups.

**Figure 8:**
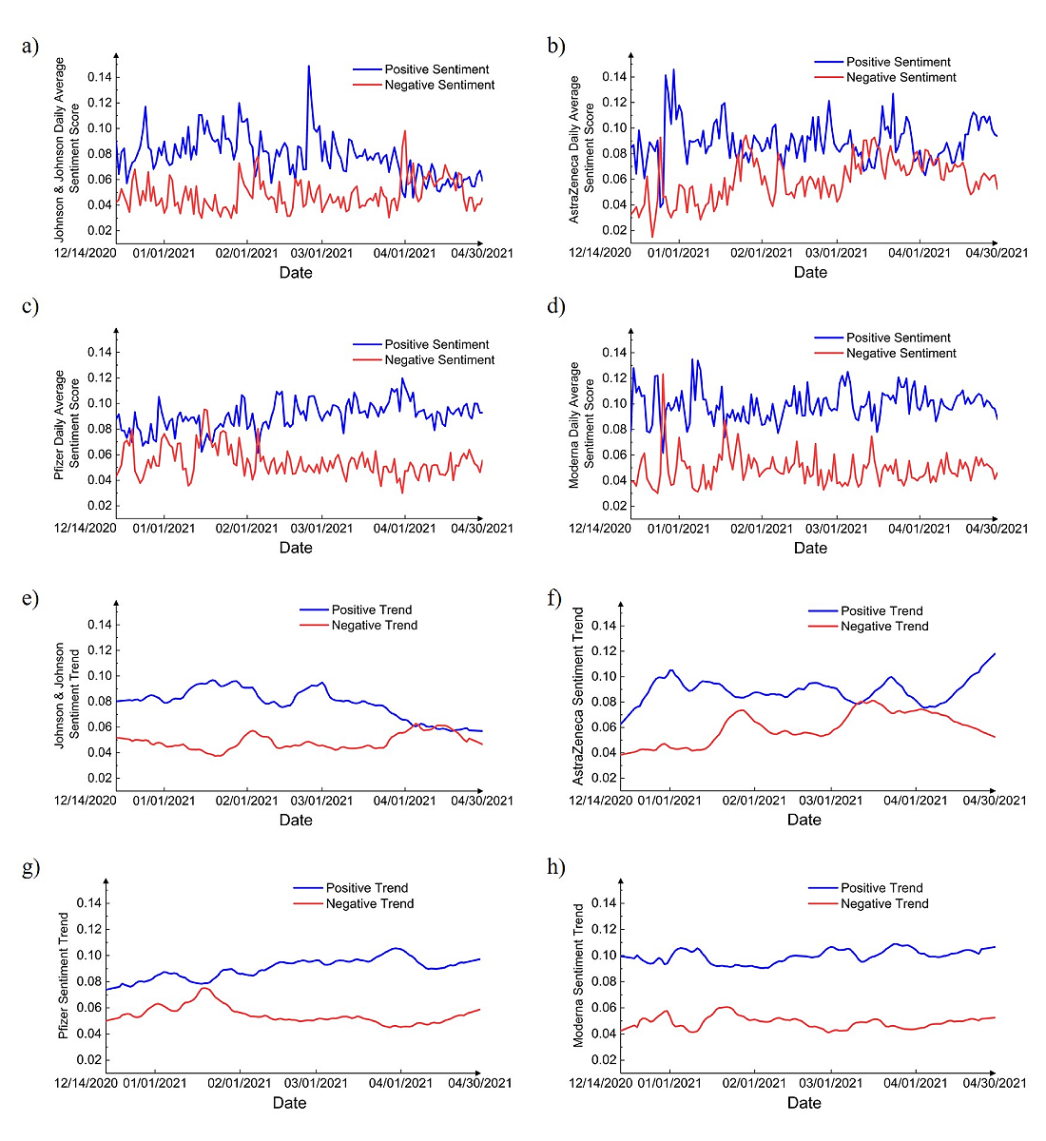
Daily average positive and negative sentiment scores for (a) Johnson & Johnson, (b) AstraZeneca, (c) Pfizer, and (d) Moderna vaccines and sentiment trends for (e) Johnson & Johnson, (f) AstraZeneca, (g) Pfizer, and (h) Moderna vaccines.

Figure 8 shows that positive sentiment and negative sentiment scores changed daily for each vaccine and positive sentiment was stronger than negative sentiment; however, for Johnson & Johnson and AstraZeneca vaccines, the average positive and negative curves were found to intersect frequently, and the differences were small. From March 11 to March 16, 2021, distribution of the AstraZeneca vaccine was suspended in Europe [29]; however, on March 18, 2021, use of the AstraZeneca vaccine resumed in Europe after a review was conducted by the European Medicines Agency [30], which may be why positive and negative sentiment curves intersect in March 2021 and positive sentiment increased soon afterward. On April 13, 2021, FDA and CDC paused the use of the Johnson & Johnson vaccine due to several reports claimed that Johnson & Johnson might be linked to a very rare serious type of blood clotting in the vaccinated individuals. This explains why the negative sentiment trend increased and positive sentiment trend decreased in April 2021, even surpassing that of positive sentiments. On April 23, 2021, the FDA and CDC lifted the pause, but the positive trend was stable and remained low, which reflected the public’s concerns about the Johnson & Johnson and AstraZeneca vaccines.

For Pfizer and Moderna vaccines, positive and negative sentiment curves were found to intersect only in December 2020 and January 2021, and the sentiment trends were stable, which reflected public concerns in the beginning, when the vaccines were first approved, followed by increasing levels of confidence in the vaccines as more and more people became vaccinated.

**Figure 9:**
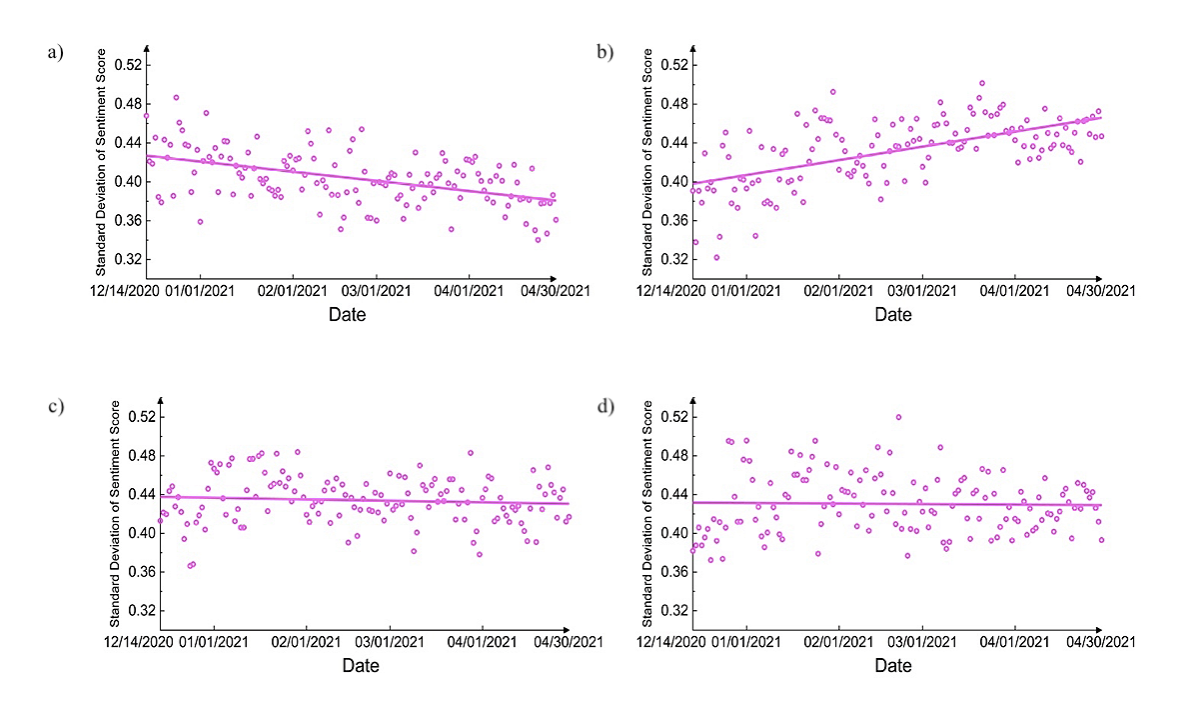
Daily standard deviation of sentiments for (a) Johnson & Johnson, (b) AstraZeneca, (c) Pfizer, and (d) Moderna vaccines.

Figure 9 shows the standard deviation of sentiments for each vaccine. For the Pfizer and Moderna vaccines, the standard deviation lines are flat, which means that the sentiments for these vaccines were very stable and did not exhibit much fluctuation. However, for Johnson & Johnson and AstraZeneca vaccines, the standard deviation of sentiments changed drastically over time. For instance, the standard deviation of the Johnson & Johnson vaccine decreased, implying a higher degree of consensus regarding this specific vaccine. However, the opposite was true for the AstraZeneca vaccine, and the increased sentiment variation indicated the attitudes toward it were found to be more divided over time.

**Figure 10:**
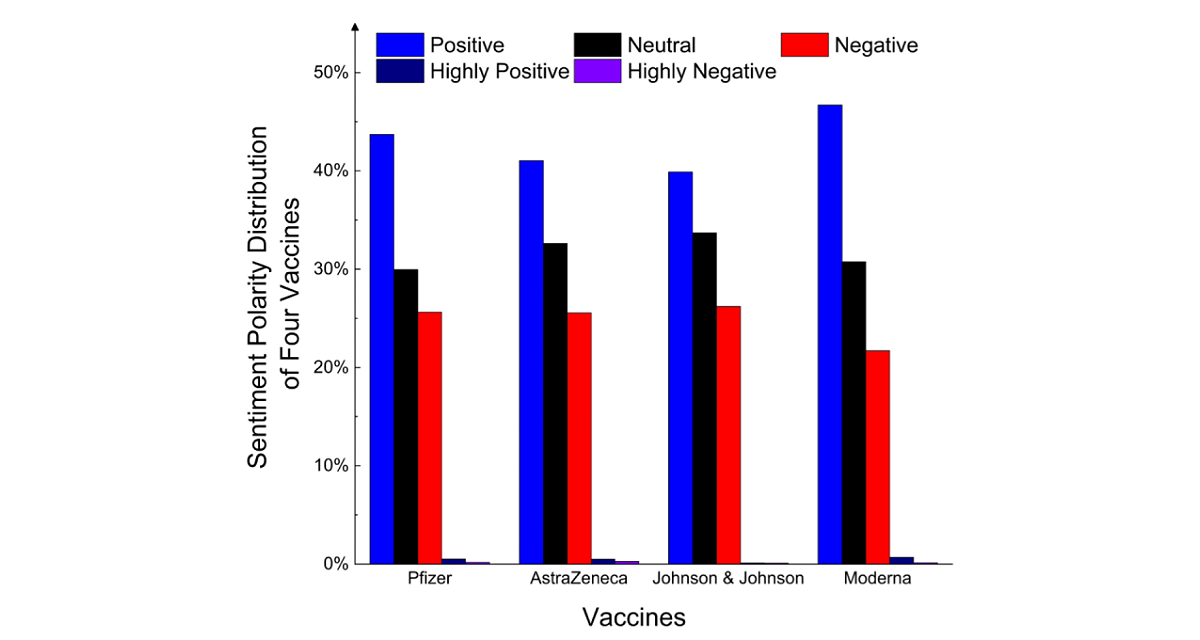
Sentiment polarity distributions for Pfizer, AstraZeneca, Johnson & Johnson, and Moderna vaccines.

Figure 10 shows the percentages of tweets for each vaccine in each sentiment polarity; the percentages in each sentiment group are very close to each other.

### Topic Modeling

#### Positive Topics

Topics suggested that people felt happy and grateful that a vaccine had been approved (Table 3), that it is important to get vaccinated, that they were thankful to the health care staff for their efforts, and that they were waiting to be eligible for vaccination.

**Table 3:** Top 5 positive (including highly positive) topics.

Topic ID	Tweets, n (%)	Keywords	Topic
POS_05	251,979 (62.13)	people, take, say, make, go, good, need, help, well, give	Planning for getting vaccination
POS_07	76,029 (18.75)	get, today, dose, first, feel, shoot, day, second, shot, be	Getting vaccinated
POS_09	21,127 (5.21)	share, read, important, health, join, question, public, information, community, concern	Vaccine information and knowledge
POS_11	14,286 (3.52)	thank, clinic, staff, support, team, volunteer, work, process, amazing, effort	Thanks for healthcare worker
POS_01	6,963 (1.72)	effective, risk, variant, pause, blood_clot, virus, benefit, less, rare, infection	Side effects

#### Neutral Topics

The main neutral topics were vaccination appointment (79,710/245,976, 32.41%) and getting vaccinated (40,532/245,976, 16.48%) (Table 4). Even though the topics were neutral, they revealed favorable attitudes toward COVID-19 vaccines. In addition, 12.77% (31,409/245,976) of neutral tweets demonstrated that people felt some hesitancy toward receiving the vaccine or that they need more time to think and make a decision.

**Table 4:** Top 5 neutral topics.

Topic ID	Tweets, n (%)	Keywords	Topic
NEU_05	79,710 (32.41)	get, today, appointment, shoot, available, be, call, wait, come, schedule	Vaccination appointment
NEU_02	40,532 (16.48)	dose, first, receive, second, shot, pfizer, day, week, administer, fully	Getting vaccinated
NEU_09	31,409 (12.77)	say, take, go, people, time, still, need, rare, would, think	Vaccine hesitancy
NEU_03	17,156 (6.97)	update, read, find, late, live, news, check, watch, question, link	Vaccine news
NEU_06	17,129 (6.96)	may, start, age, year, week, open, next, eligible, site, begin	Vaccine eligibility

#### Negative Topics

Negative topics (Table 5) demonstrated the public’s main concerns regarding COVID-19 vaccines. In general, the public mainly cared about the side effects of vaccines, including common side effects, such as soreness after receiving a vaccine, and serious adverse reactions, such as death. However, given the strict storage requirement, the vaccines’ supply chain and rollout were the second most important issue that concerned the public. Other negative topics involved the vaccination appointment, coronavirus variants, vaccination for women and patients with cancer (people who are at high risk), fake news, and misinformation.

**Table 5:** Negative (including highly negative) topics.

Topic ID	Tweets, n (%)	Keywords	Topics
NEG_05	115,206 (56.04)	get, people, take, go, say, make, know, stop, need, still	Vaccine hesitancy
NEG_00	19,690 (9.58)	risk, death, case, report, blood_clot, rare, severe, low, receive, blood	Extreme side effects
NEG_06	17,154 (8.34)	government, country, pay, company, rollout, state, plan, fail, stock, supply	Vaccine supply and rollout
NEG_04	14,125 (6.87)	get, shoot, feel, arm, day, hour, today, shot, sore, second	Common side effects
NEG_07	10,248 (4.98)	appointment, wait, available, age, site, open, today, hospital, group, offer	Vaccination appointment
NEG_03	8080 (3.93)	use, emergency, say, suspend, break, astrazeneca, official, country, shortage, pause	AstraZeneca suspension
NEG_02	7100 (3.45)	dose, week, first, second, receive, next, day, ruin, delay, administer	Vaccine administration
NEG_09	6151 (2.99)	read, question, health, public, story, information, hesitancy, register, community, explain	Vaccine information and community
NEG_01	4471 (2.17)	pandemic, virus, new, fight, variant, lockdown, avoid, coronavirus, spread, restriction	Spread avoidance
NEG_08	3367 (1.64)	cause, cancer, clot, woman, trust, product, doctor, body, choice, damage	Extreme side effects on vulnerable groups

We found that 47.32% of the tweets (405,560/857,128), demonstrated positive (including highly positive) attitudes toward COVID-19 vaccines. The main topics included encouraging people to get vaccinated and conveying hope and gratitude for future life as a result of vaccine approval. Overall, 23.99% of the tweets (205,592/857,128) expressed negative (including highly negative) attitudes and concerns. The main concerns regarding COVID-19 vaccines were side effects of vaccination, serious adverse reactions, and vaccine supply.

#### Topic Evolution

**Figure 11:**
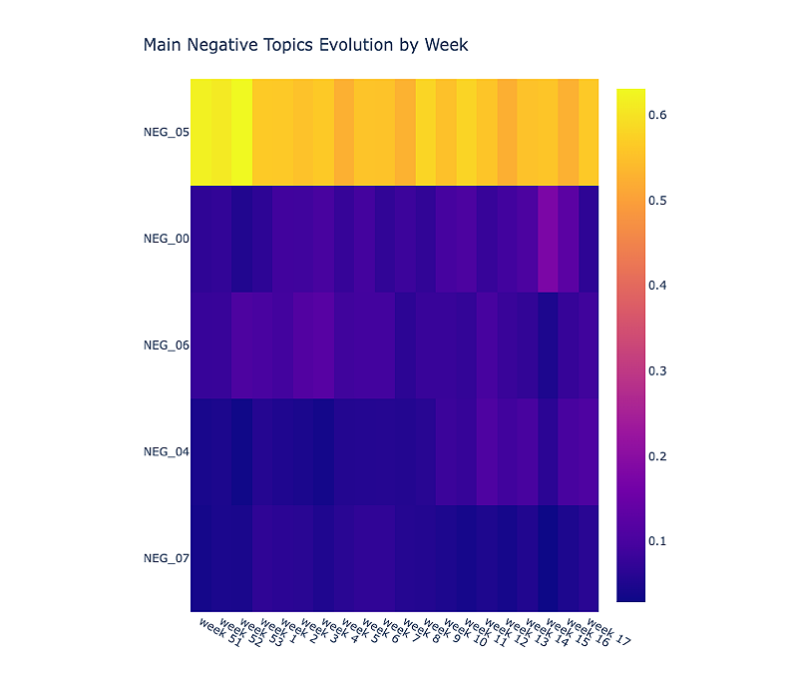
Heatmap of negative topic evolution. The x-axis represents the week in the year. Lighter colors correspond to topics that are discussed more.

Side effects, such as pain at the injection site (ie, NEG_05) were discussed the most (of all negative topics) throughout the period (Figure 11). Moreover, with the increase in the number of people who received the vaccine, the discussion on side effects increased. Topics such as vaccine supply (ie, NEG_00) and extreme side effects (ie, NEG_06) were discussed less but a consistent amount throughout the period.

## Discussion

### General Sentiments

Most sentiments toward COVID-19 vaccines were neutral and positive. Positive sentiment was stronger than negative sentiment throughout the period. Previous results from research conducted from March 1 to November 22, 2020 (before vaccines were available) [3] were similar—the dominant sentiments were positive and neutral; however, in this study, negative sentiment (205,592/857,128, 23.99%) was lower than that in [3] (30.57%). This suggests that after the COVID-19 vaccines became available, their effectiveness in reducing the risk of infection started to manifest in the real world, and people started having fewer doubts on social media toward vaccines. Vaccine trials, social media, and government interventions may contribute to alleviating public concerns [31].

### Concerns and Topics That Shape Attitudes

By applying topic modeling to our data set, we found that the main topic in the positive and neutral domain was encouraging people to get vaccinated. In general, we discovered that vaccines are becoming widely accepted by the public as time passes. The main topic of our negative data set was the severe side effects of vaccination. When some social media outlets reported possible vaccination side effects, the concerns were discussed frequently on different social media platforms, such as Twitter, and possibly impacted individual decisions. Before vaccines were available, discussions on vaccines were centered around clinical trials and vaccine availability [12]. However, upon vaccine rollout, the concerns shifted dramatically to common side effects, which dominated the discussion throughout the study period (from December 14, 2020 to April 30, 2021). Hence, timely monitoring of the public attitude can help guide public health officials to communicate more effectively with the public.

We also found that among the negative tweets, other than vaccine hesitancy, the main concerns regarding side effects (NEG_00 and NEG_04) were vaccine supply and rollout (NEG_06). This finding is consistent with those from previous studies [24,32,33]. For example, in a study on vaccination hesitancy in Canada [24], it was found that vaccination hesitancy stemmed from mistrust toward vaccine development, lack of knowledge about COVID-19 vaccines, and suspicion about political and authority figures who were not taking the vaccine. In another study [32] employing a questionnaire for the Israeli population, the results showed that the top 3 concerns regarding COVID-19 vaccines were quality control, side effects, and doubtful efficiency. Another survey conducted in the United States and Canada showed that vaccine rejection is very strongly related to vaccine benefits, vaccine safety, and unforeseen future effects [33]. Overall, our findings were similar—the top concerns were vaccine safety, side effects, vaccine supply, and government policy.

### Changes by Month

Overall, it was observed that positive sentiment distribution decreased, neutral sentiment distribution increased, and negative sentiment distribution was stable. However, positive sentiment was dominant throughout the study period (December 14, 2020 to April 30, 2021). Positive sentiment decreased in March and April 2021, likely because of the extreme side effects (blood clotting) reported in the news for Johnson & Johnson and AstraZeneca vaccines. Use of the AstraZeneca vaccine was even stopped in Europe briefly [29], and the FDA and CDC called for a pause on the use of the Johnson & Johnson vaccine in the United States [28]. This may have caused positive sentiment to decrease, while neutral sentiment rather than negative sentiment increased, because people tended to feel neutral rather than very negative, toward such a pause.

In the very beginning, such side effects were extensively discussed. Some news outlets reported severe side effects, such as Bell palsy and even death [34], after receiving the vaccine, which seemed to coincide with more negative sentiments. Both Pfizer and Moderna vaccines are mRNA vaccines, which is a new type of vaccine that has not been used before [35]. This caused the general public to have concerns regarding the long-term side effects of these novel vaccines [7]. In the beginning, the lack of knowledge about COVID-19 and mRNA vaccines shaped the public’s concerns. However, as more people were vaccinated over time, more people were able to observe how these vaccines helped steadily decrease the number of new cases and deaths per day as well as the hospitalization rates, implying that the pandemic is under control thanks to these vaccines. This in turn resulted in an increasing number of people seeking to become vaccinated, because extreme side effects are very rare and might be associated with misinformation and because the common side effects are regarded as tolerable.

Sentiment trend findings were consistent with those from a previous study [22] in which a vaccine acceptance experiment using Weibo Sina (a popular social media platform in China) demonstrated that positive attitudes were dominant, that the Chinese population were inclined to be positive about the side effects over time, and that one of the concerns that affects vaccine acceptance are misunderstandings about vaccination.

### Limitations and Future Work

In this study, we mainly focused on textual information from the Twitter platform. However, users may be distributed among different social media platforms and different locations according to their usage, language, and preferences. Therefore, the methods used in our study can be extended to different social media platforms. It is also possible to use geographical filters on location information or to work on other languages to precisely differentiate between the significant issues and concerns among the different cultures or demographics.

Furthermore, our model can be extended to other research problems. For example, future studies should focus on negative tweets to determine whether misinformation exists or to identify misinformation on social media and propose suggestions for how to minimize the spread of such misinformation. Moreover, it may be plausible in the future to train a topic model with LDA and deep learning to forecast event topics and trends.

### Conclusions

Our work profiles the spectrum of public sentiments toward vaccination and the main concerns underlying these views since the rollout of vaccines. These findings demonstrate the effectiveness of sentiment–based topic modeling in identifying topics and trends in polarity groups and in revealing the dynamic nature of public attitudes toward vaccination in the midst of evolving situations and changing public measures during the pandemic. Adding sentiment analysis and topic modeling when monitoring COVID-19 vaccine awareness can help researchers uncover time–based viewpoints underlying the dynamic public attitude toward vaccination on a large scale and devise tailored communication strategies to promote vaccination.

## Appendix

Multimedia Appendix 1 Related work on sentiment analysis or topic modeling.

## Abbreviations

CDC: Centers for Disease Control
FDA: US Food and Drug Administration
LDA: latent Dirichlet allocation
VADER: Valence Aware Dictionary for Sentiment Reasoning

## References

[1] (2021). COVID-19 vaccines are effective. Centers for Disease Control and Prevention.

[2] D'Souza G, Dowdy D Rethinking herd immunity and the covid-19 response endgame. Johns Hopkins Bloomberg School of Public Health.

[3] Hussain A, Tahir A, Hussain Z, Sheikh Z, Gogate M, Dashtipour K, Ali A, Sheikh A (2021). Artificial intelligence-enabled analysis of public attitudes on Facebook and Twitter toward covid-19 vaccines in the United Kingdom and the United States: observational study. J Med Internet Res.

[4] Lazarus JV, Ratzan SC, Palayew A, Gostin LO, Larson HJ, Rabin K, Kimball S, El-Mohandes A (2021). A global survey of potential acceptance of a covid-19 vaccine. Nat Med.

[5] Leischow SJ, Milstein B (2006). Systems thinking and modeling for public health practice. Am J Public Health.

[6] Romer D, Jamieson KH (2020). Conspiracy theories as barriers to controlling the spread of COVID-19 in the U.S. Soc Sci Med.

[7] Hitti FL, Weissman D (2021). Debunking mRNA vaccine misconceptions-an overview for medical professionals. Am J Med.

[8] Infodemic. World Health Organization.

[9] Eysenbach Gunther (2020). How to fight an infodemic: the four pillars of infodemic management. J Med Internet Res.

[10] Serrano P, Huangfu L (2021). CURVE4COVID: comprehensive understanding via representative variable exploration for COVID-19. Proceedings of 2021 Americas Conference on Information Systems.

[11] Li Xiaoming, Zeng Wenbing, Li Xiang, Chen Haonan, Shi Linping, Li Xinghui, Xiang Hongnian, Cao Yang, Chen Hui, Liu Chen, Wang Jian (2020). CT imaging changes of corona virus disease 2019(COVID-19): a multi-center study in Southwest China. J Transl Med.

[12] Wen Andrew, Wang Liwei, He Huan, Liu Sijia, Fu Sunyang, Sohn Sunghwan, Kugel Jacob A, Kaggal Vinod C, Huang Ming, Wang Yanshan, Shen Feichen, Fan Jungwei, Liu Hongfang (2021). An aberration detection-based approach for sentinel syndromic surveillance of COVID-19 and other novel influenza-like illnesses. J Biomed Inform.

[13] Li L, Zhang Q, Wang X, Zhang J, Wang T, Gao T, Duan W, Tsoi Kk, Wang F (2020). Characterizing the propagation of situational information in social media during COVID-19 epidemic: a case study on Weibo. IEEE Trans Comput Soc Syst.

[14] Chew Cynthia, Eysenbach Gunther (2010). Pandemics in the age of Twitter: content analysis of Tweets during the 2009 H1N1 outbreak. PLoS One.

[15] Signorini Alessio, Segre Alberto Maria, Polgreen Philip M (2011). The use of Twitter to track levels of disease activity and public concern in the U.S. during the influenza A H1N1 pandemic. PLoS One.

[16] Mutanga Mb, Abayomi A (2020). Tweeting on COVID-19 pandemic in South Africa: LDA-based topic modelling approach. Africa J Sci Technol Innov Dev.

[17] Oyebode Oladapo, Ndulue Chinenye, Adib Ashfaq, Mulchandani Dinesh, Suruliraj Banuchitra, Orji Fidelia Anulika, Chambers Christine T, Meier Sandra, Orji Rita (2021). Health, Psychosocial, and Social Issues Emanating From the COVID-19 Pandemic Based on Social Media Comments: Text Mining and Thematic Analysis Approach. JMIR Med Inform.

[18] Jang Hyeju, Rempel Emily, Roth David, Carenini Giuseppe, Janjua Naveed Zafar (2021). Tracking COVID-19 Discourse on Twitter in North America: Infodemiology Study Using Topic Modeling and Aspect-Based Sentiment Analysis. J Med Internet Res.

[19] Garcia Klaifer, Berton Lilian (2021). Topic detection and sentiment analysis in Twitter content related to COVID-19 from Brazil and the USA. Appl Soft Comput.

[20] S.V. P, Ittamalla R. (2021). An analysis of attitude of general public toward COVID-19 crises – sentimental analysis and a topic modeling study. Inf Discov Deliv.

[21] Abdulaziz M, Alotaibi A, Alsolamy M, Alabbas A (2021). Topic based sentiment analysis for covid-19 tweets. Int J Adv Comput Sci Appl.

[22] Yin Fulian, Wu Zhaoliang, Xia Xinyu, Ji Meiqi, Wang Yanyan, Hu Zhiwen (2021). Unfolding the determinants of covid-19 vaccine acceptance in China. J Med Internet Res.

[23] Hou Zhiyuan, Tong Yixin, Du Fanxing, Lu Linyao, Zhao Sihong, Yu Kexin, Piatek Simon J, Larson Heidi J, Lin Leesa (2021). Assessing covid-19 vaccine hesitancy, confidence, and public engagement: a global social listening study. J Med Internet Res.

[24] Griffith Janessa, Marani Husayn, Monkman Helen (2021). COVID-19 vaccine hesitancy in Canada: content analysis of tweets using the theoretical domains framework. J Med Internet Res.

[25] Hutto C., Gilbert E. (2014). VADER: a parsimonious rule-based model for sentiment analysis of social media text. Proceedings of the International AAAI Conference on Web and Social Media.

[26] Jelodar H, Wang Y, Yuan C, Feng X, Jiang X, Li Y, Zhao L (2021). Latent Dirichlet allocation (LDA) and topic modeling: models, applications, a survey. arXiv.

[27] Blei D M, Ng A Y, Jordan M I (2003). Latent Dirichlet allocation. J Mach Learn Res.

[28] US Food and Drug Administration.

[29] AstraZeneca shares slide as clotting reports lead Denmark to pause rollout of its vaccine. Fortune.

[30] AstraZeneca’s COVID vaccine gets all-clear from EU health agency following blood clot uproar. Fortune.

[31] Bavel Jay J Van, Baicker Katherine, Boggio Paulo S, Capraro Valerio, Cichocka Aleksandra, Cikara Mina, Crockett Molly J, Crum Alia J, Douglas Karen M, Druckman James N, Drury John, Dube Oeindrila, Ellemers Naomi, Finkel Eli J, Fowler James H, Gelfand Michele, Han Shihui, Haslam S Alexander, Jetten Jolanda, Kitayama Shinobu, Mobbs Dean, Napper Lucy E, Packer Dominic J, Pennycook Gordon, Peters Ellen, Petty Richard E, Rand David G, Reicher Stephen D, Schnall Simone, Shariff Azim, Skitka Linda J, Smith Sandra Susan, Sunstein Cass R, Tabri Nassim, Tucker Joshua A, Linden Sander van der, Lange Paul van, Weeden Kim A, Wohl Michael J A, Zaki Jamil, Zion Sean R, Willer Robb (2020). Using social and behavioural science to support COVID-19 pandemic response. Nat Hum Behav.

[32] Dror Amiel A, Eisenbach Netanel, Taiber Shahar, Morozov Nicole G, Mizrachi Matti, Zigron Asaf, Srouji Samer, Sela Eyal (2020). Vaccine hesitancy: the next challenge in the fight against COVID-19. Eur J Epidemiol.

[33] Taylor Steven, Landry Caeleigh A, Paluszek Michelle M, Groenewoud Rosalind, Rachor Geoffrey S, Asmundson Gordon J G (2020). A proactive approach for managing covid-19: the importance of understanding the motivational roots of vaccination hesitancy for SARS-CoV2. Front Psychol.

[34] Buntz Brian (2021). Is there a link between Bell’s palsy and COVID-19 vaccines?. Drug Discovery Trends.

[35] Beyrer Chris (2021). The long history of mRNA vaccines. Johns Hopkins Bloomberg School of Public Health.

