# Correction: COVID-19 Vaccine Tweets After Vaccine Rollout: Sentiment–Based Topic Modeling

**DOI:** 10.2196/37841

**Published:** 2022-03-11

**Authors:** Luwen Huangfu, Yiwen Mo, Peijie Zhang, Daniel Dajun Zeng, Saike He

**Affiliations:** 1 Fowler College of Business San Diego State University San Diego, CA United States; 2 Center for Human Dynamics in the Mobile Age San Diego State University San Diego, CA United States; 3 The State Key Laboratory of Management and Control for Complex Systems Institute of Automation Chinese Academy of Sciences Beijing China; 4 University of Chinese Academy of Sciences Beijing China

In “COVID-19 Vaccine Tweets After Vaccine Rollout: Sentiment–Based Topic Modeling” (J Med Internet Res 2022;24(2):e31726) the authors noted one error.

In the originally published paper, Figure 8D showed incorrect colors. The top line was intended to be blue, and the bottom line was intended to be red.

In the corrected version of the paper, Figure 8D has been revised as follows: the top line is now blue, and the bottom line is now red. The correct figure is provided below. The originally published Figure 8 is in Multimedia Appendix 1.

The correction will appear in the online version of the paper on the JMIR Publications website on March 11, 2022, together with the publication of this correction notice. Because this was made after submission to PubMed, PubMed Central, and other full-text repositories, the corrected article has also been resubmitted to those repositories.

**Figure 8:**
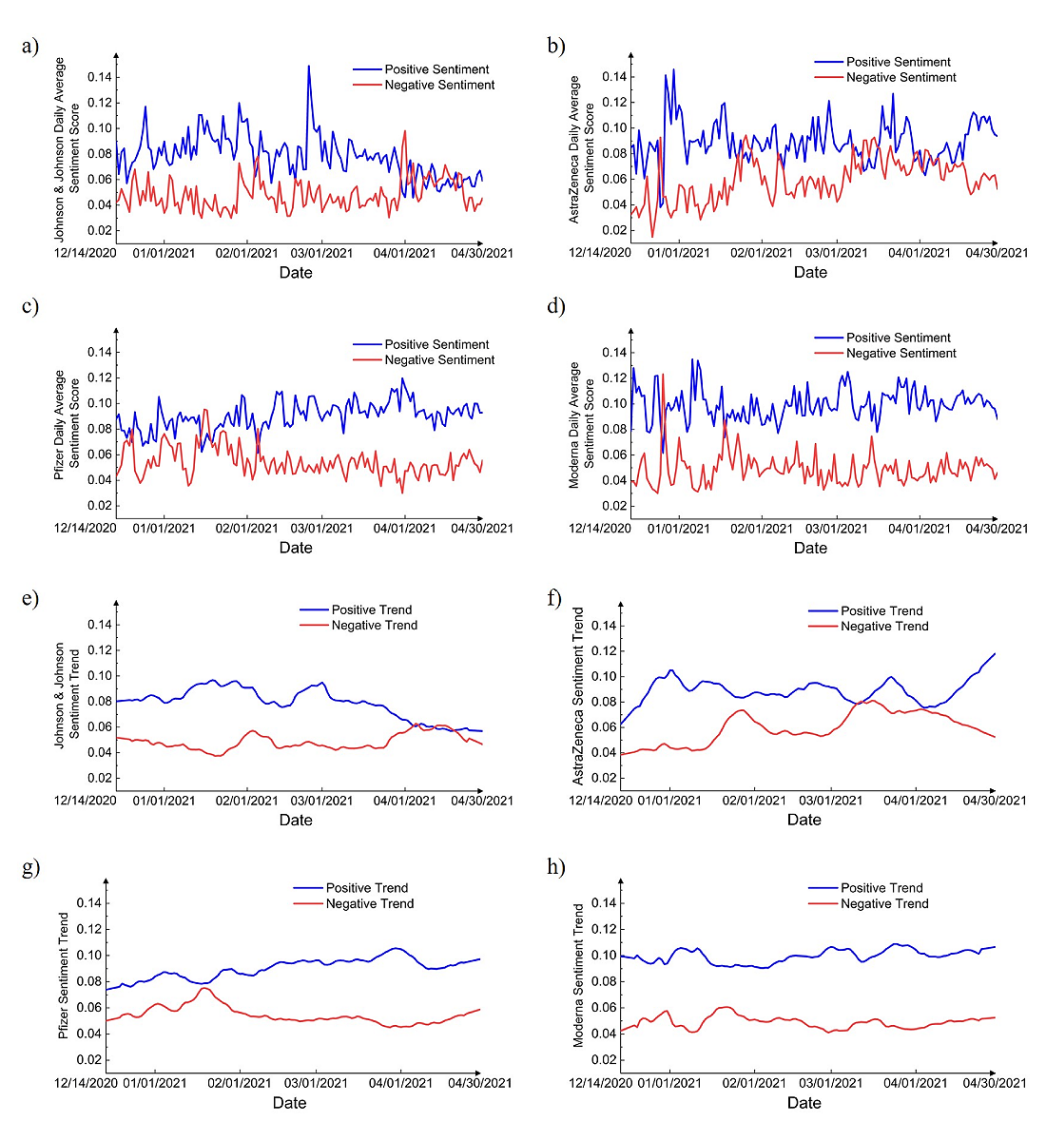
Daily average positive and negative sentiment scores for (a) Johnson & Johnson, (b) AstraZeneca, (c) Pfizer, and (d) Moderna vaccines and sentiment trends for (e) Johnson & Johnson, (f) AstraZeneca, (g) Pfizer, and (h) Moderna vaccines.

## Appendix

Multimedia Appendix 1 The previously published Figure 8.

