# Supplementary material for: COVID-19 Vaccine Tweets After Vaccine Rollout: Sentiment–Based Topic Modeling
Source: J Med Internet Res. 2022 Feb 8;24(2):e31726. doi: 10.2196/31726 (PMC8827037; doi:10.2196/31726)
Supplement: Multimedia Appendix 1 [file jmir_v24i2e31726_app1.docx]

**Table S1.** Related work on sentiment analysis or topic modeling on COVID-19 related data.

| Authors | Vaccine-specific | Source | Language | Sentiment Analysis | Topic Modeling | Large-Scale/  Automatic Processing | Time | After Vaccine Rollout^[[1]](#footnote-1)^ |
| --- | --- | --- | --- | --- | --- | --- | --- | --- |
| Mutanga & Abayomi [16] | ✗ | Twitter | Not specified | ✗ | ✓ | ✓ | 3/15/2020 to 4/30/2020 | ✗ |
| Oyebode et al.[17] | ✗ | Twitter, YouTube, Facebook, PushSquare.com, Archinect.com, and LiveScience.com | English | ✓ | ✓ | ✓ | Not specified | ✗ |
| Jang et al. [18] | ✗ | Twitter | English | ✓ | ✓ | ✓ | 1/28/2020 to 5/11/2020 | ✗ |
| Garcia & Berton[19] | ✗ | Twitter | Portuguese & English | ✓ | ✓ | ✓ | 4/17/2020 to 8/8/2020 | ✗ |
| S.V. ^[[2]](#footnote-2)^ & Ittamalla [20] | ✗ | Twitter | Not specified | ✓ | ✓ | ✓ | 2/1/2020 to 6/27/2020 | ✗ |
| Abdulaziz et al.[21] | ✗ | Twitter | English | ✓ | ✓ | ✓ | 3/1/2020 to 4/30/2020 &  9/1/2020 to 10/31/2020 | ✗ |
| Hussain et al.[3] | ✓ | Twitter, Facebook | English | ✓ | ✗ | ✓ | 3/1/2020 to 11/22/2020 | ✗ |
| Hou et al.[23] | ✓ | Twitter, Sina Weibo | Not specified | ✓ | ✗ | ✗ | 6/13/2020 to 7/31/2020 | ✗ |
| Yin et al. [22] | ✓ | Sina Weibo | Chinese | ✓ | ✓ | ✓ | January to October 2020 | ✗ |
| Griffith et al.[24] | ✓ | Twitter | English | ✗ | ✓ | ✗ | 12/10/2020 to 12/23/2020 | ✓ |
| *Our study* | *✓* | *Twitter* | *English* | *✓* | *✓* | *✓* | *12/14/2020 to 4/30/2021* | *✓* |

1. Even though this information can be inferred from the “Time” column, we add it here for better understanding the time sequence with vaccine rollout. [↑](#footnote-ref-1)
2. This is indeed the first author’s last name. We have doubled checked about this to make sure. [↑](#footnote-ref-2)
