# Supplementary figures and images for: Correction: COVID-19 Vaccine Tweets After Vaccine Rollout: Sentiment–Based Topic Modeling
Source: J Med Internet Res. 2022 Mar 11;24(3):e37841. doi: 10.2196/37841 (PMC8956994; doi:10.2196/37841)

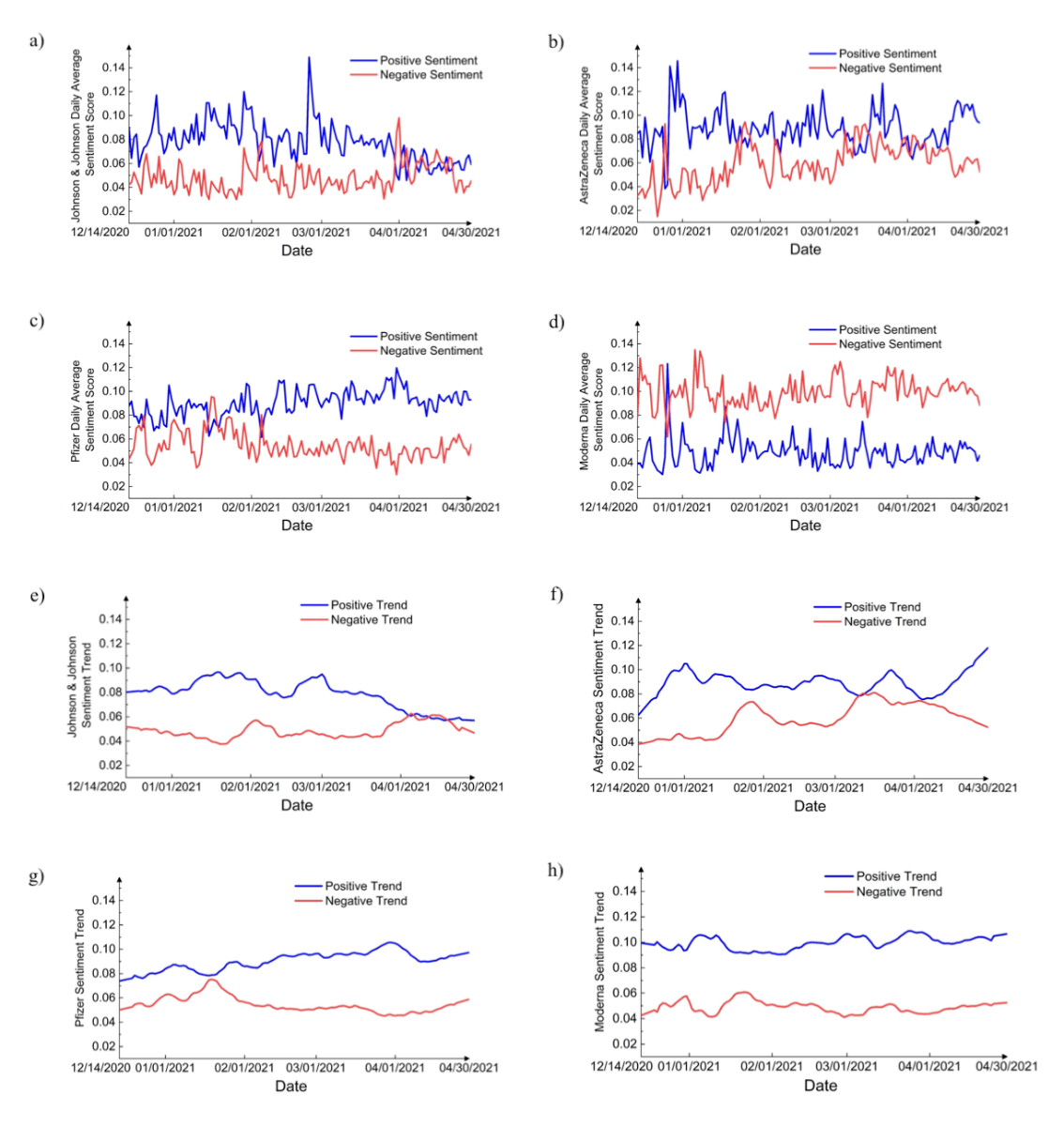

Supplement: Multimedia Appendix 1 [file jmir_v24i3e37841_app1.png]
